# The utility of lung epithelium specific biomarkers in cardiac surgery: a comparison of biomarker profiles in on- and off-pump coronary bypass surgery

**DOI:** 10.1186/1749-8090-8-4

**Published:** 2013-01-09

**Authors:** Gerwin E Engels, Y John Gu, Willem van Oeveren, Gerhard Rakhorst, Massimo A Mariani, Michiel E Erasmus

**Affiliations:** 1 HaemoScan B.V, Groningen, The Netherlands; 2 Department of Cardiothoracic Surgery, University of Groningen, University Medical Center Groningen, Groningen, The Netherlands; 3 Department of Surgery, University of Groningen, University Medical Center Groningen, Groningen, The Netherlands

**Keywords:** Clara cell 16 kD protein, Surfactant protein D, Cardiopulmonary bypass, Off-pump, CABG

## Abstract

**Background:**

Despite continuous improvements in materials and perfusion techniques, cardiac surgery still causes lung injury and a delay of pulmonary recovery. Currently, there is no gold standard for quantifying cardiac surgery induced lung injury and dysfunction. Adding objective measures, such as plasma biomarkers, could be of great use here. In this study the utility of lung epithelium specific proteins as biomarkers for lung dysfunction was evaluated.

**Methods:**

Serial measurements of plasma concentrations of Clara cell 16 kD (CC16) protein, Surfactant protein D (SP-D), Elastase and Myeloperoxidase were performed on blood samples from 40 patients who underwent coronary artery bypass grafting with cardiopulmonary bypass (CABG, n = 20) or without cardiopulmonary bypass (OPCAB, n = 20).

**Results:**

The increase of SP-D and CC16 between pre-operative concentrations and concentrations at the end of cardiopulmonary bypass, correlated with the Aa-O_2_ gradient at 1 hour on the ICU (R_s_ = 0.409, *p* = .016 and R_s_ = 0.343, *p* = .043, respectively).

Furthermore, SP-D and CC16 were higher in CABG than in OPCAB at the end of surgery [8.96 vs. 4.91 ng/mL, *p* = .042 and 92 vs. 113%, *p* = .007, respectively]. After 24 h both biomarkers returned to their baseline values.

**Conclusions:**

Our results show that increases in plasma of SP-D and CC16 correlate with clinical lung injury after coronary artery bypass surgery. Therefore, lung epithelium specific proteins seem to be a useful biomarker for measuring lung injury in the setting of cardiac surgery.

## Background

Despite continuous improvements in materials and perfusion techniques, cardiac surgery still causes lung injury and a delay of pulmonary recovery [1,2]. This delay can partly be attributed to the unique aspects of cardiac surgery, such as the sternotomy, cardioplegia, and the use of cardiopulmonary bypass (CPB). Pulmonary dysfunction following cardiac surgery varies between hypoxemia to acute respiratory distress syndrome. Currently, there is no gold standard for quantifying cardiac surgery induced lung injury and dysfunction. A vast series of physiological changes (alveolar-arterial oxygen pressure difference, lung compliance, pulmonary vascular resistance, etc.) and measurement of lung unspecific inflammation markers such as neutrophil elastase, and myeloperoxidase [1] have been reported.

The use of lung epithelium specific secretory proteins for evaluating the integrity of the alveolar capillary membrane has been proposed as an alternative method to assess lung injury [3]. For instance, variations in plasma concentrations of surfactant proteins (surfactant protein A and D) were associated with sepsis, respiratory distress syndrome and interstitial lung diseases [4]. Recently, a surfactant protein has been used as a marker for lung injury after surgery with CPB [5].

Clara cells, mainly located in the (terminal) bronchioles, are responsible for protecting the bronchiolar epithelium, by detoxifying inhaled substances and secreting the anti-inflammatory Clara Cell 16 kD protein (CC16) [6]. Serum concentrations of CC16 have been associated with injury of the alveolar-capillary membrane, and are nowadays often used as a biomarker of injury to the alveolar-capillary membrane in different models [7,8].

The utility of these lung epithelium specific proteins as biomarkers for lung dysfunction in the setting of cardiac surgery is unknown. To explore this, we performed serial measurements of surfactant protein D and CC16 in a patient group undergoing elective coronary bypass surgery either with or without the use of cardiopulmonary bypass. It was expected that the use of CPB during coronary bypass surgery would result in more lung dysfunction [9], and consequently higher plasma concentrations of lung epithelium specific proteins. Secondly, the aim was to analyse if there was a correlation between these lung epithelium specific proteins and lung dysfunction. Lung dysfunction was assessed by the PaO_2_/FiO_2_ ratio and the alveolar-arterial oxygen pressure gradient (Aa-O_2_ gradient) on the ventilator on the intensive care unit (ICU).

## Methods

### Study subjects and design

Patient material from a previous study investigating the role of CPB on RBC aggregation and deformability was used [10]. Forty patients with indication for coronary surgery were prospectively included after approval from the local institutional review board and informed consent from each individual. Patients were randomly allocated to a group operated with cardiopulmonary bypass (CABG, n = 20) or without (OPCAB, n = 20). The inclusion criterion for the study was first time CABG. Exclusion criteria were emergency surgery, significantly impaired ventricular function (EF <35%) or a previous cerebrovascular accident.

For all patients, anaesthesia was induced and maintained by the intravenous infusion of midazolam and sufentanil followed by a median sternotomy. In the CABG group, CPB was performed with a heart–lung machine consisting of roller pumps and a membrane oxygenator with integrated heat exchanger. During CPB, moderate hypothermia (34°C) was applied with a pump flow of 2.4 L/min/m^2^. Whole body anticoagulation was achieved with 300 IU/ (kg body weight) heparin in CABG patients and 100 IU/ (kg body weight) heparin in OPCAB patients.

For patients undergoing CABG, blood samples of 10 mL were taken immediately after the induction of anaesthesia but before surgery (PRE-OP), 5 minutes after start of surgery (START-OP), 5 minutes after the whole body heparinisation (HEPARIN), 5 minutes after start of CPB and haemodilution (START CPB/30’ HEP), 15 minutes after end of CPB (END CPB/ANASTOMOSIS), one hour after surgery (1 h ICU), and on the first postoperative morning (24 h ICU). For the OPCAB patients, the sampling time was similar to the CABG patients, except for START CPB/30’ HEP and END CPB/ANASTOMOSIS, which were taken 30 minutes after heparinisation and 15 minutes after the end of coronary anastomosis, respectively. Blood gas samples were taken one hour after surgery (1 h ICU). Each blood sample was anticoagulated with 0.1 mM EDTA. Plasma was obtained by centrifugation of whole blood at 1100 × g for 10 min. Hereafter, plasma was aliquoted and stored at −80°C for later analysis.

### Determination of surfactant protein D

Surfactant protein D plasma concentration as a marker of alveolar-capillary membrane integrity was measured in plasma by means of sandwich ELISA. Capture and detection antibodies were from R&D Systems (R&D Systems, Minneapolis, USA). Recombinant human Surfactant protein D (R&D Systems, Minneapolis, USA) served as a standard. Inter- and intra-assay coefficient of variation were 4.4% and 2.6%, respectively.

### Determination of Clara cell 16 kD protein

Clara cell 16 protein (CC16), a marker of respiratory epithelial integrity, was measured by sandwich ELISA. Recombinant human CC16 (R&D Systems, Minneapolis, USA) served as a standard. A monoclonal rat antibody to human CC16 (R&D Systems, Minneapolis, USA) was used as a capture antibody and monoclonal mouse antibody to human CC16 (Hycult, Uden, The Netherlands) was used as a detection antibody. Inter- and intra-assay coefficient of variation were 5.6% and 4.8%, respectively.

### Determination of elastase-α_1_-antitrypsin complex

Elastase plasma concentration as a marker of leukocyte activation was determined by means of sandwich ELISA. Antibodies were purchased from Affinity Biologicals (Affinity Biologicals Inc., Ancaster, Canada). Elastase isolated from human donor leukocytes (Merck KGaA, Darmstadt, Germany) served as a standard. Inter- and intra-assay coefficient of variation were 6.3% and 5.2%, respectively.

### Determination of myeloperoxidase (MPO)

MPO plasma concentration as another marker of leukocyte activation was also determined by means of sandwich ELISA. Capture and detection antibodies were purchased from Hytest (HyTest LTD, Turku, Finland). Myeloperoxidase isolated from human donor leukocytes (HyTest LTD, Turku, Finland) served as a standard. Inter- and intra-assay coefficient of variation were 8.6% and 6.5%, respectively.

### Data analysis

Biochemical measurements were corrected for hemodilution by using hematocrit levels. All values are summarized as mean and standard deviation, or median and interquartile range in case the data were non-normally distributed. The Student’s t-test was used to compare means of continuous variables, whereas the Mann–Whitney U test was used for non-normally distributed variables. Correlations were assessed with Spearman rank correlation tests. Contingency tables, χ^2^ and Fisher exact tests were used as appropriate. A two-way mixed ANOVA was used to compare serial data. Violations of sphericity were Greenhouse-Geisser corrected. All tests performed in order to test the (null-) hypothesis of no difference were two-sided. A *p*-value < .05 was considered statistically significant. Statistical analyses were performed with SPSS version 16.0 for Windows (SPSS Inc., Chicago, Ill, United States).

## Results

Forty patients scheduled for elective cardiac surgery were included in this study. Patient characteristics are described in Table 1. There were no significant differences between the groups with regard to relevant baseline characteristics, except for the number of diabetic patients in the OPCAB group, which was less than in the CABG group.

**Table 1:** Baseline characteristics and operative variables

**Variable**	**CABG (n = 20)**	**OPCAB (n = 20)**	***p *value**^**a**^
Baseline characteristics			
Age [years]			0.857
Mean	64	63	
SD	9	11	
Length [cm]			0.487
Mean	174	172	
SD	6	8	
Weight [kg]			0.536
Mean	81	79	
SD	11	12	
Gender [No.]			0.168
men	16 (80%)	12 (60%)	
Coexisting illness [No.]			
Diabetes	5	0	0.047
Pulmonary disease	3	1	0.605
Postoperative variables			
Operation time [min]			< 0.001
Mean	205	126	
SD	31	49	
Cross clamb time [min]			
Mean	51	-	
SD	16	-	
CPB time [min]			
Mean	81	-	
SD	22	-	
Creatinine clearance [μmol/L]			0.668
Mean	76.3	79.2	
SD	14.6	25.3	
Ventilation time [h]			0.001
Mean	13.7	5.1	
SD	7.5	7.1	
PaO2/FiO2 [mmHg]			0.064
Median	189	238	
IQR	163 - 264	214 - 272	
Aa-O2 gradient [mmHg]			0.015
Median	159	137	
IQR	137 - 172	116 - 153	

^a^ Student’s t-test, Mann-Whtiney U test, χ2 or Fisher excact test used as appropriate.

**Figure 1:**
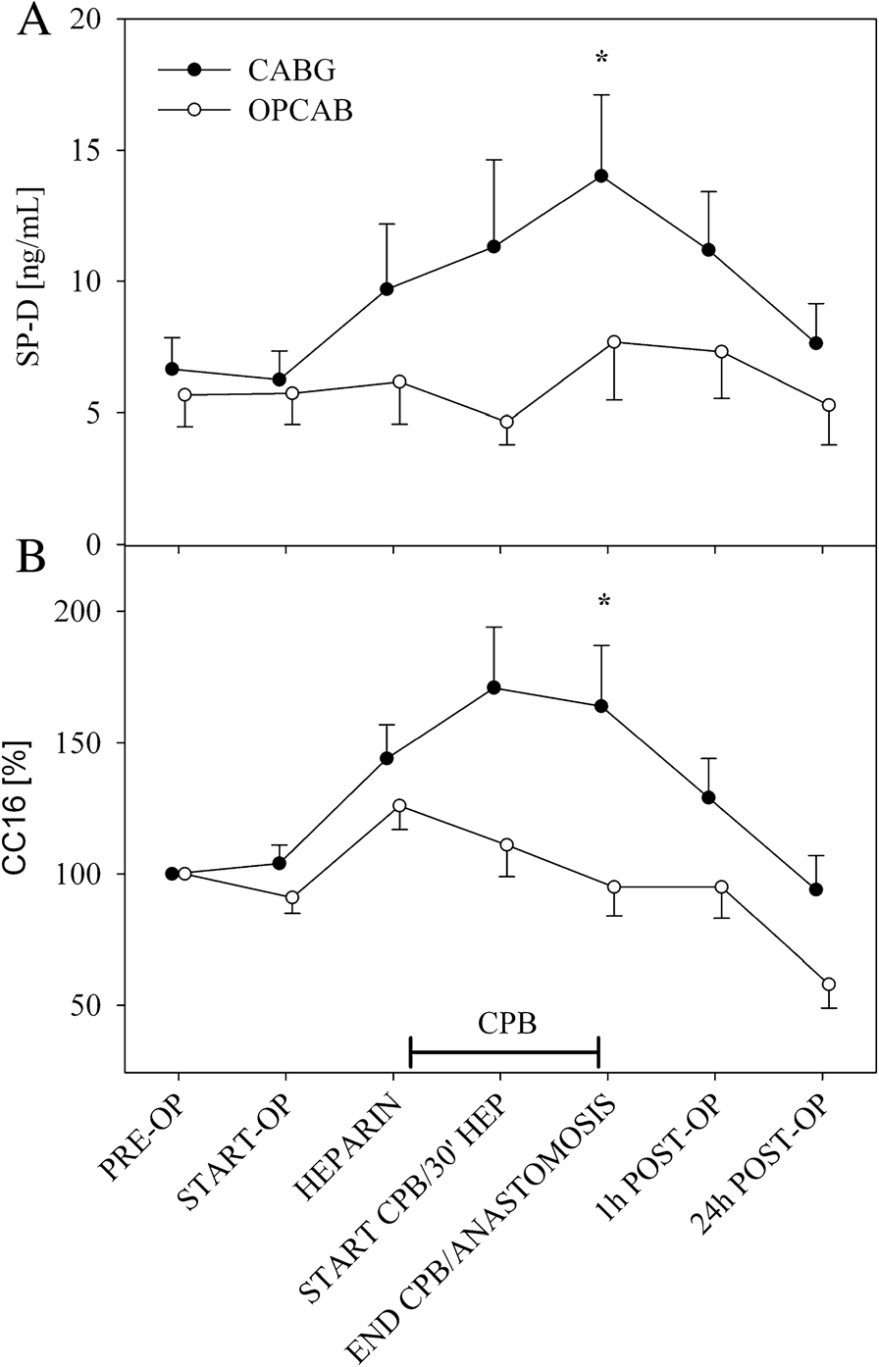
**A) Surfactant protein D plasma concentrations.** Data shown are mean ± SEM of SP-D plasma concentrations measured by ELISA in CABG and OPCAB patients (Overall *p* = .014). **B**) CC16 plasma concentrations. Data shown are mean ± SEM of CC16 plasma concentrations measured by ELISA in CABG and OPCAB patients (Overall *p* = .068). Closed and open circles represent CABG and OPCAB, respectively. * *p* < .05 CABG versus OPCAB.

In the CABG group, SP-D plasma concentration (Figure 1A) was significantly increased at the end of surgery (END CPB/ANASTOMOSIS) as compared with the OPCAB group [8.96 vs. 4.91 ng/mL (median), *p* = .042]. One day after surgery (24 h ICU), SP-D plasma concentration returned to baseline values. In the OPCAB group median SP-D plasma concentration remained constant during the procedure.

To compare between groups, interindividual differences in CC16 plasma concentration made it necessary to normalize CC16 values by means of the baseline measurement: CC16_i_normalized_ = CC16_i_ / CC16_Baseline_ × 100%. Data before normalization are summarized in Table 2. During cardiopulmonary bypass CC16 plasma concentration increased in the CABG group whereas there was no increase in the OPCAB group (Figure 1B). Plasma concentrations returned to baseline values on the first postoperative day (24 h ICU).

**Table 2:** Biomarker plasma concentrations

									***p *values**^**a**^	
**Variable**	**Pre-op**	**Start-op**	**Heparin**	**Start CPB/30’ Hep**	**End CPB/Anastomosis**	**1 h ICU**	**24 h ICU**	**Between groups**	**Between timepoints**	**Interaction**
**SP-D [ng/mL] - Median (IQR)**								0.068	0.001	0.014
CABG	4.15 (2.88-7.99)	4.71 (2.83-7.92)	4.59 (3.00-13.36)	6.20 (3.19-13.44)	8.96 (7.32-14.12)	8.71 (5.25-13.91)	5.69 (2.99-11.46)			
OPCAB	4.39 (1.97-8.08)	3.51 (2.67-6.95)	4.27 (2.61-6.75)	4.14 (2.48-5.26)	4.91 (2.07-10.72)	5.08 (3.43-7.39)	3.99 (0.59-6.72)			
**CC16 [ng/mL] - Median (IQR)**								0.988	<0.001	0.068
CABG	5.41 (4.38-9.21)	6.45 (4.25-9.45)	8.15 (5.88-13.56)	10.36 (6.18-16.79)	10.47 (6.50-14.34)	7.95 (4.33-13.23)	4.66 (3.92-9.18)			
OPCAB	5.14 (5.59-15.06)	7.95 (4.79-11.09)	10.61 (7.62-14.02)	8.33 (6.22-11.64)	8.34 (5.64-13.42)	7.15 (4.57-14.50)	3.92 (2.28-7.43)			
**Elastase [**μ**g/mL] - Median (IQR)**								0.002	<0.001	<0.001
CABG	2.16 (1.63-3.19)	1.87 (1.61-3.46)	2.68 (2.03-3.13)	4.09 (2.32-4.95)	29.1 (14.3-2.24)	37.7 (14.0-55.9)	16.7 (7.63-24.6)			
OPCAB	1.43 (1.17-2.85)	2.17 (1.61-3.46)	2.12 (1.25-2.86)	2.89 (2.12-3.47)	4.02 (2.24-4.90)	5.13 (3.13-6.95)	12.9 (7.97-20.9)			
**MPO [ng/mL] - Median (IQR)**								0.025	<0.001	<0.001
CABG	37 (28–45)	28 (23–48)	441 (334–527)	507 (401–571)	191 (133–310)	134 (80–170)	52 (45–58)			
OPCAB	41 (32–50)	38 (32–45)	367 (314–386)	360 (319–388)	172 (101–304)	109 (64–148)	59 (48–80)			

IQR, inter quartile range; ^a^ Two-way mixed ANOVA.

Elastase was measured as an index of leukocyte activation (Table 2). After reperfusion (END CPB/ANASTOMOSIS) elastase markedly increased in the CABG group and was significantly higher than in the OPCAB group [29.1 vs. 4.0 μg/mL (median), *p* < .001]. At one hour after surgery (1 h ICU) this difference was even larger [37.7 vs 5.1 μg/mL (median), *p* < .001]. On the first postoperative day (24 h ICU) elastase plasma concentration in the CABG group returned to baseline whereas it was moderately increased in the OPCAB group.

Myeloperoxidase concentration, another maker for leukocyte activation, increased in both groups after whole body heparinisation (Table 2), however MPO plasma concentration was significantly higher in the CABG group as compared with the OPCAB group [507 vs. 360 ng/mL (median), *p* = .002]. One day after surgery MPO plasma concentration returned to baseline values in both groups.

Two-way mixed ANOVA revealed an overall increase for SP-D, MPO and Elastase in the CABG group as compared to the OPCAB group (*p* = .014, *p* < .001 and *p* < .001, respectively), CC16 also showed an increase although not statistically significant (*p* = .068).

At 1 hour after surgery (1 h ICU) lung function as assessed by PaO_2_/FiO_2_ was better in the OPCAB group as compared to the CABG group (Table 1). Analogously, the Aa-O_2_ gradient was significantly lower in the OPCAB group. Our data show an association between the net increase in SP-D (ΔSP-D, SP-D at 1 h ICU minus SP-D at PRE-OP) and PaO_2_/FiO_2_ (Table 3). Similarly, there is an association between ΔSP-D and the Aa-O_2_ gradient.

**Table 3:** **Correlation between lung biomarkers and PaO**_**2**_**/FiO**_**2 **_**and Aa-O**_**2**_** gradient at 1 h ICU**

	**PaO**_**2**_**/FiO**_**2**_**[mmHg]**		**Aa-O**_**2**_**gradient [mmHg]**	
	**Rs**	***p*-value**	**Rs**	***p*-value**
**SP-D [ng/mL]**	0.462	.006	0.409	.016
**CC16 [%]**	0.342	.044	0.343	.043

The normalized values of CC16 at the end of cardiopulmonary bypass (END CPB/ANASTOMOSIS) depicted a similar association with PaO_2_/FiO_2_ (Table 3) and the Aa-O_2_ gradient.

**Figure 2:**
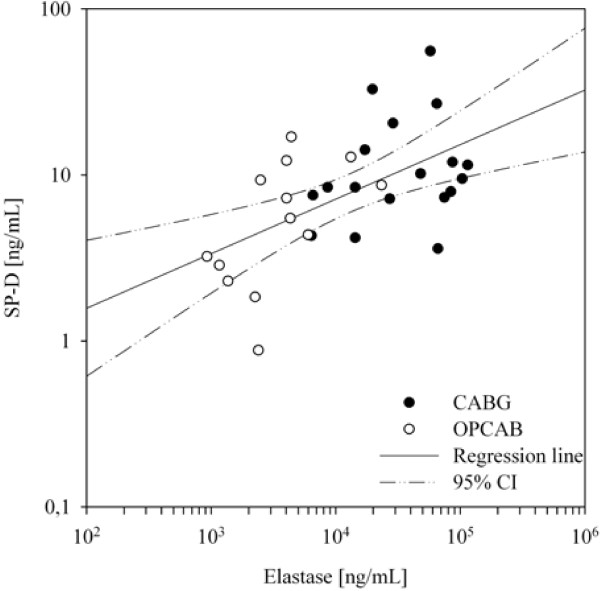
**Correlation between Elastase [ng/mL] and SP-D [ng/mL] in CABG and OPCAB patients immediately following reperfusion (END CPB/ANASTOMOSIS).** Spearman’s rho = 0.548, *p* < .001.

Since SP-D and Elastase showed similar trends during the coronary bypass surgery procedure, their relation after reperfusion (END CPB/ANASTOMOSIS) was analysed. The two variables exhibited a significant association (Figure 2, Spearman’s rho = 0.548, *p* = .001).

## Discussion

This study shows that SP-D and CC16 are sensitive markers of lung injury and that they can be useful in the setting of cardiac surgery. After CABG, with or without CPB, higher SP-D and CC16 plasma concentrations were associated with more lung injury as assessed by the PaO_2_/FiO_2_ ratio and the Aa-O_2_ gradient early on the ICU. Secondly, higher SP-D and CC16 plasma concentrations after CABG with CPB as compared to OPCAB were found.

The use of lung epithelium specific proteins for describing lung injury is attractive because there are hardly any direct lung injury markers available. To our knowledge only the group of Boven *et al*. has utilised this before by measuring CC16 and KL-6 (Krebs von den Lungen-6) for comparing standard CPB with a mini extracorporeal circuit [11]. They found that CC16 is a potential biomarker for damage to the alveolar capillary membrane during coronary artery bypass surgery.

There are several hypotheses for explaining the increase of lung epithelium specific proteins, 1) increased permeability of the alveolar capillary membrane, 2) increased production or secretion of these proteins, or 3) decreased renal clearance [12].

The increase in elastase plasma concentration, and its association with ΔSP-D, supports the notion of increased permeability of the alveolar capillary membrane after CPB, a well-described phenomenon caused by an inflammation reaction induced by CPB. This reaction includes activation of the coagulation cascade, the complement system and release of cytokines and adhesion receptors which results in neutrophil-endothelial cell interactions that liberate lung macrophage proteases and neutrophilic enzymes, such as elastase, and produce diffuse tissue injury and increased pulmonary vascular permeability [2,13]. It is well known that elastase has multiple effects on the respiratory epithelium; one of them is the reduction in integrity of the epithelium by cleaving E-cadherin [14]. Furthermore, the increase in elastase represents leukocyte activation, which also results in generation and release of reactive oxygen species [15]. Reactive oxygen species contribute to a decrease in pulmonary endothelial barrier function by disrupting intercellular tight junctions and redistribution of focal adhesions [16].

Another explanation for higher lung epithelium specific proteins levels might be an increased production or secretion of these proteins in the bronchioli and alveoli after CPB, suggesting a protective function of these lung proteins. This is supported by a three-fold increase of SP-B and SP-C in tracheal aspirates after surgery with CPB in children [17]. Literature does not provide reference values for CC16 in bronchoalveolar lavage fluid (BALF) after cardiac surgery. However, Determan *et al*. analysed CC16 plasma and BALF concentrations following various surgical procedures to compare the influence of two mechanical ventilation strategies. The authors compared CC16 concentrations directly after intubation and after 5 h of surgery and they found an increase in plasma concentrations but not in BALF concentrations [18]. This supports the explanation of increased permeability rather than increased production or secretion.

Whether higher concentrations of lung epithelium specific proteins in the epithelium lining fluid lead to higher plasma concentrations without an increased permeability is not clear. Perhaps only a larger concentration gradient between alveoli and circulation is sufficient for increasing plasma concentrations. Our finding that plasma concentrations of lung epithelium specific proteins returned to baseline 24 h after surgery (paralleled by elastase plasma concentrations) suggests a temporary increase in permeability of the alveolar capillary membrane.

The third possible explanation for increased plasma concentration of lung epithelium specific proteins is decreased renal function. Although there is always some degree of decreased renal function following CABG, about 7% decrease in creatinine clearance in a recent study [19], this small decrease is not likely responsible for the increase in SP-D and CC16. Moreover, we found no difference in creatinine clearance between groups, but we did find higher concentrations of lung epithelium specific proteins in the CABG group.

Our second finding was the reduction of lung epithelium specific proteins in the OPCAB group. OPCAB is known to reduce the inflammatory reaction as compared to CABG with CPB [20,21]. Therefore, it may be expected that OPCAB can reduce the occurrence of diffuse tissue injury and a reduced leakage of lung epithelium specific proteins.

The benefits of preventing clinical lung injury and/or dysfunction by OPCAB are still debated; there are studies that report better gas exchange after OPCAB [22] and others that do not [23-25]. These results are based on clinical parameters such as the gradient between inspired oxygen concentration and arterial blood oxygen tension. Possibly these parameters are not sensitive enough to detect injury to the alveolar capillary membrane in low risk patients with relatively short operation times. The use of lung epithelium specific biomarkers can be of use, as we were able to show an increase in SP-D and CC16 plasma concentrations in the CABG group. This may explain the finding that OPCAB significantly reduced pulmonary complications such as ventilation time and pneumonia and also a shorter ICU stay and 30 day mortality in a recent study by others [26].

Myeloperoxidase, which is considered as a measure for the activation of neutrophils, increased right after the administration of heparin. This profile of MPO during CPB surgery has been reported before [27], and it can be explained by heparin-induced liberation of endothelial bound MPO [28]. Moreover, the liberation of MPO seems to be dose dependent as the CABG group reveived three times more heparin than the OPCAB group. Altogether, these observations suggest that MPO might not be a good biomarker for activation of neutrophils in the setting of CPB.

## Conclusions

We have shown that higher SP-D and CC16 plasma concentrations correlate with more clinical lung injury after coronary bypass surgery. Furthermore, the CABG group had higher SP-D and CC16 plasma concentrations and more lung injury as compared with the OPCAB group. Lung epithelium specific proteins seem to be a useful biomarker for measuring lung injury and can be used for assessing strategies meant to reduce CPB-induced lung injury.

## Competing interests

The authors declare that they have no competing interests.

## Authors’ contributions

YG, WvO and ME conceived and designed the study. GE performed the measurements, analysis and drafted the manuscript. All authors were involved in the interpretation of the data, revised the article critically, and gave their final approval of the version to be published.
